# Establishment of Chronic Typhoid Infection in a Mouse Carriage Model Involves a Type 2 Immune Shift and T and B Cell Recruitment to the Gallbladder

**DOI:** 10.1128/mBio.02262-19

**Published:** 2019-10-01

**Authors:** Juan F. González, Jonathan Kurtz, David L. Bauer, Regan Hitt, James Fitch, Amy Wetzel, Krista La Perle, Peter White, James McLachlan, John S. Gunn

**Affiliations:** a Center for Microbial Pathogenesis, The Research Institute at Nationwide Children’s Hospital, Columbus, Ohio, USA; b Department of Pediatrics, College of Medicine, The Ohio State University, Columbus, Ohio, USA; c Infectious Diseases Institute, The Ohio State University, Columbus, Ohio, USA; d Department of Microbial Infection and Immunity, The Ohio State University, Columbus, Ohio, USA; e The Institute for Genomic Medicine, The Research Institute at Nationwide Children’s Hospital, Columbus, Ohio, USA; f Department of Veterinary Biosciences, Comparative Pathology and Mouse Phenotyping Shared Resource, The Ohio State University, Columbus, Ohio, USA; g Department of Microbiology & Immunology, Tulane University Health Sciences Center, New Orleans, Louisiana, USA; Harvard Medical School

**Keywords:** *Salmonella*, biofilm, chronic carriage

## Abstract

Typhoid fever, caused primarily by Salmonella enterica serovar Typhi (*S*. Typhi), is a life-threatening systemic disease responsible for significant morbidity and mortality worldwide. Three to 5% of individuals infected with *S*. Typhi become chronic carriers due to bacterial persistence in the gallbladder. We have demonstrated that *Salmonella* forms biofilms on gallstones to establish gallbladder carriage. However, an in-depth molecular understanding of chronic carriage in the gallbladder, from the perspective of both the pathogen and host, is poorly defined. To examine the dynamics of the gallbladder in response to *Salmonella* infection, we performed transcriptional profiling in the mouse gallbladder at early (7 days) and chronic (21 days) time points. Transcriptome sequencing (RNA-Seq) revealed a shift from a Th1 proinflammatory response at 7 days postinfection (dpi) toward an anti-inflammatory Th2 response by 21 dpi, characterized by increased levels of immunoglobulins and the Th2 master transcriptional regulator, GATA3. Additionally, bioinformatic analysis predicted the upstream regulation of characteristic Th2 markers, including interleukin-4 (IL-4) and Stat6. Immunohistochemistry and fluorescence-activated cell sorter (FACS) analysis confirmed a significant increase in lymphocytes, including T and B cells, at 21 dpi in mice with gallstones. Interestingly, the levels of *Salmonella*-specific CD4 T cells were 10-fold higher in the gallbladder of mice with gallstones at 21 dpi. We speculate that the biofilm state allows *Salmonella* to resist the initial onslaught of the Th1 inflammatory response, while yet undefined events influence a switch in the host immunity toward a more permissive type 2 response, enabling the establishment of chronic infection.

## Abstract (precis)

The existence of chronic typhoid carriers has been in the public eye for over 100 years in part because of the publicity around Typhoid Mary. Additionally, it has been known for decades that the gallbladder is the main site of persistence and recently that gallstones play a key role. Despite this, very little is known about the physiological conditions that allow Salmonella enterica serovar Typhi to persist in the gallbladder. In this study, we analyze the transcriptional profile of the gallbladder in a mouse model of chronic carriage. We found a shift from an early proinflammatory immune response toward a later anti-inflammatory response, which could explain the stalemate that allows *Salmonella* persistence. Interestingly, we found a 10-fold increase in the number of *Salmonella*-specific T cells in mice with gallstones. This work moves us closer to understanding the mechanistic basis of chronic carriage, with a goal toward eradication of the disease.

## INTRODUCTION

Typhoid fever, caused primarily by Salmonella enterica serovar Typhi (*S.* Typhi), is a life-threatening systemic disease that is responsible for significant morbidity and mortality annually worldwide (1). Approximately 3 to 5% of individuals infected with *S.* Typhi become chronic carriers, who are typically asymptomatic and can spread the disease through fecal shedding. The chronic carrier state is associated with *Salmonella* colonization of the biliary tract and is positively correlated with cholelithiasis, with up to 90% of carriers having gallstones (2). *S*. Typhi is a human-restricted pathogen; therefore, asymptomatic carriers represent a critical reservoir for further spread of disease. We have previously demonstrated that gallstones aid in the development and maintenance of gallbladder carriage in a mouse model of *Salmonella* infection, as well as in humans, where gallstones serve as a substrate to which salmonellae attach and form a protective biofilm (3, 4).

The immune response to *Salmonella* systemic acute infection has been widely studied. *Salmonella* is transmitted through the fecal-oral route and, once it reaches the intestines, invades the host through M cells in the Peyer’s patches. Subsequently, typhoidal strains, including S. enterica serovar Typhimurium in the mouse, can spread systemically via the lymphatic system and replicate within phagocytic cells in the liver, spleen, and bone marrow (5–7). CD4^+^ T cells recognize major histocompatibility complex (MHC)-presented bacterial antigens and are an essential defense against *Salmonella*. Importantly, the development of Th1 cells and the release of gamma interferon (IFN-γ), IL-12, and tumor necrosis factor alpha (TNF-α) has been shown to be crucial for controlling bacterial growth and for effective clearance (8, 9). B cells are important for acquired immunity, not by the production of antibodies as they are expendable against secondary *Salmonella* infection, but instead for the priming of *Salmonella*-specific Th1 cells (10). IL-17-producing Th17 cells are also involved by recruiting neutrophils to combat infection (11). Less is known about immunity in the hepatobiliary system, especially the response to infection in the gallbladder and whether this immune response inhibits or contributes to carriage of bacteria.

The molecular basis of chronic carriage of *Salmonella* in the gallbladder, from both the host and bacterial perspectives, is poorly understood but displays similar characteristics to other biofilm-associated chronic diseases (12). This led us to investigate the special conditions that allow *Salmonella* to persist in the gallbladder environment. We developed a gallstone mouse model using *S.* Typhimurium to mimic human chronic carriage (4). We have previously found that cholelithiasis induced by a lithogenic diet causes pronounced inflammation in the biliary tract (cholecystitis) in mice (13). These observations led us to hypothesize that, during cholelithiasis, *Salmonella* biofilms on gallstones promote a permissive immune environment that allows for the establishment of chronic infection. To test this hypothesis, we examined the transcriptome of the mouse gallbladder at 7 and 21 days postinfection (dpi) with *Salmonella* and followed this by directly assessing the immune cell populations present in the gallbladder at 21 dpi.

## RESULTS

### Transcriptomic analysis of the cholecystitis gallbladder after *Salmonella* infection.

**FIG 1:**
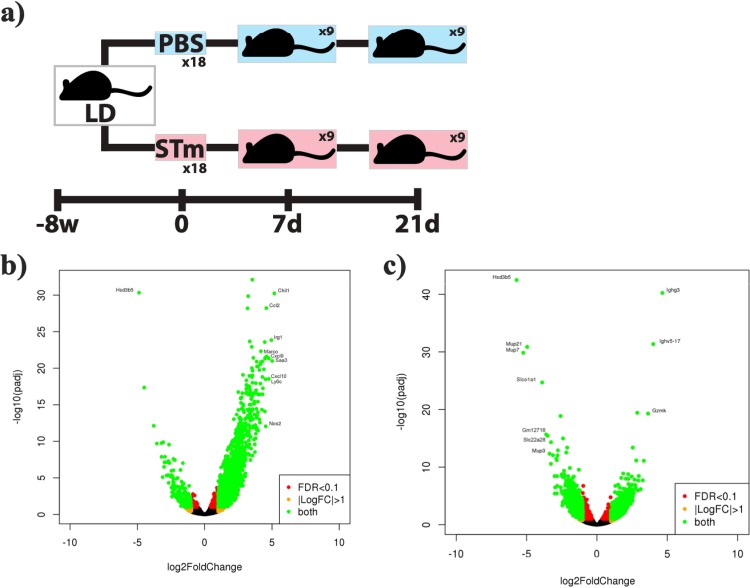
(a) Experimental setup of the chronic carriage mouse model. All mice received a lithogenic diet: half were infected with 1 × 10^4^ *S.* Typhimurium (STm) cells, and half were mock infected with PBS. Gallbladders were removed at 7 and 21 dpi, total RNA was isolated, and RNA-Seq was performed. (b and c) Volcano plots of differentially expressed genes. The *x* axis specifies the log_2_ fold changes (FC), and the *y* axis specifies the negative logarithm to the base 10 of the test *P* values. Green points represent genes that meet the filtering criteria (FC ≥ 2, *P *≤ 0.05; DESeq2): 1,650 at 7 dpi (b) and 1,402 at 21 dpi (c).

Starting with the hypothesis that an altered immune response during *Salmonella* gallbladder colonization allows the bacterium to establish a chronic infection, we set out to elucidate the transcriptional profile of the gallbladder using our chronic carriage mouse model at two time points: first, an early time point of acute disease at 7 dpi where the gallbladder shows visual signs of inflammation, and a later time point at 21 dpi, where we have previously observed signs of gallbladder epithelium and lamina propria tissue repair (14). For this set of experiments, all mice received a lithogenic diet, and mock-infected mice were injected with phosphate-buffered saline (PBS) as a vehicle control for both time points (Fig. 1a).

As expected, *Salmonella* infection had large effect on gene expression at 7 dpi (see Table S1 in the supplemental material). In total, 1,650 genes were differentially expressed, with a majority of genes upregulated at 7 dpi (Fig. 1b), using a fold change (FC) of ≥2 and a *P* value of ≤0.05 as a cutoff. As can be observed in Fig. 1b, most of the differentially expressed genes were upregulated at 7 dpi (i.e., most green points were on the right side). There was a clear overrepresentation of immune response genes (Table 1). The top upregulated gene was *Chil1* (FC = 36.16), which codes for a highly conserved protein that binds chitin but has no catalytic activity and plays a critical role in pathogen response (15–17). Other highly upregulated loci include immune response genes like *Ly6c1*, *Saa3*, *Irg1*, *Marco*, *Nos2*, and various chemokine genes, including *Cxcl9*, *Ccl2*, and *Cxcl10*. Ingenuity Pathway Analysis (IPA) revealed that the most significantly represented pathway (by lowest *P* value) was Trem1 signaling with 35 upregulated genes. This pathway plays an important role in innate immune response by contributing to the activation of the inflammatory response and septic shock in response to microbial infections (18), followed by bacterial pattern recognition receptors, with 44 upregulated genes. These receptors recognize conserved microbial structures or pathogen-associated molecular patterns (PAMPs) and initiate a response (19) and communication between innate and adaptive immune cells, with 37 upregulated genes. These include genes involved in communication via dendritic cells, cytokines, and chemokines (see Table S2 in the supplemental material). A high number of downregulated genes at 7 dpi are involved in steroid metabolic process, including the top gene, *Hsd3b5*, which is part of the 3-β-HSD enzymatic system, which plays a key role in the biosynthesis of all classes of hormonal steroids (Table S1).

**TABLE 1:** Immune response-related genes upregulated in lithogenic diet mouse gallbladders after *Salmonella* infection at 7 and 21 days

Function	Product of upregulated gene at:	
	7 dpi	21 dpi
Inflammasome	IL-1β	IL-1β
	Casp1	Casp1
	NLRP3	
TLR activation and signaling	TLR1	
	TLR2	
	TLR6	
	TLR7	
	TLR8	
	TLR9	TLR9
	TLR11	
	TLR13	
	CD14	CD14
	MYD88	
Th1 T cells	CD4	
	STAT1	
	STAT4	STAT4
	TBX21 (T-bet)	TBX21 (T-bet)
	IFN-γ	
	IL-12α (p35)	
	IL-12β (p40)	IL-12β (p40)
	CCR5	
	CXCR3	
Th2 T cells		GATA3
Regulation of T cell differentiation	SOCS1	
	SOCS3	SOCS3
CD8 T cells	CD8A	CD8A
	EOMES	
	FASLG (CD95L)	FASLG (CD95L)
Tregs	FOXP3	
	TGF-β1	TGF-β1
T cell signaling and activation	CD3E	CD3E
	CD5	
	Zap70	
	CD69	CD69
	CD40L	CD40L
	ICOS	ICOS
		CD28
T cell costimulation	CD80	CD80
	CD86	
	CD40	
	ICOSL	
Dendritic cell markers	CD83	
	Batf3	
	CD8A	
	ITGAM (CD11b)	ITGAM (CD11b)
	ITGAX (CD11c)	ITGAX (CD11c)
	IGTB2 (CD11c)	
	ITGA4 (CD49d)	ITGA4 (CD49d)
	ITGAE (CD103)	

10.1128/mBio.02262-19.1 TABLE S1 Differentially expressed genes at 7 days postinfection. Shown are RNA-Seq results at 7 dpi. A total of 1,650 genes were differentially expressed, using FC ≥ 2 and *P* ≤ 0.05 as a cutoff. Genes are listed by the absolute value of the fold change. Download Table S1, XLSX file, 0.4 MB. Copyright © 2019 González et al. 2019 González et al. This content is distributed under the terms of the Creative Commons Attribution 4.0 International license.

10.1128/mBio.02262-19.2 TABLE S2 Top differentially expressed pathways at 7 days postinfection. Shown are the most significantly represented pathways predicted at 7 dpi by Ingenuity Pathway Analysis (listed by lowest *P* value). Download Table S2, XLSX file, 0.1 MB. Copyright © 2019 González et al. 2019 González et al. This content is distributed under the terms of the Creative Commons Attribution 4.0 International license.

At 21 dpi there are fewer differentially expressed genes (*n* = 1,402) (Fig. 1c; see Table S3 in the supplemental material), perhaps reflecting our previous observation that tissue morphology is returning to normal at this time. Interestingly, most of the top upregulated genes code for antibodies, indicating the presence of B cells in the gallbladder. Furthermore, another of the top upregulated genes at 21 dpi is *Gata3* (FC = 5.81, *P* < 0.001), the master regulator for Th2 T cell differentiation. The top pathways by IPA at 21 dpi were T cell receptor signaling (27 genes), lipopolysaccharide (LPS)/IL-1-mediated inhibition of retinoid X receptor (RXR) function (40 genes), iCOS-iCOSL signaling in T helper cells (25 genes), and CTLA4 signaling cytotoxic T cells (22 genes). Similar to the 7-dpi results, the 21-dpi downregulated genes were mostly involved in hormonal steroid biosynthesis (see Table S4 in the supplemental material).

10.1128/mBio.02262-19.3 TABLE S3 Differentially expressed genes at 21 days postinfection. Shown are RNA-Seq results at 21 dpi. A total of 1,402 genes were differentially expressed, using FC ≥ 2 and *P* ≤ 0.05 as a cutoff. Genes are listed by the absolute value of the fold change. Download Table S3, XLSX file, 0.2 MB. Copyright © 2019 González et al. 2019 González et al. This content is distributed under the terms of the Creative Commons Attribution 4.0 International license.

10.1128/mBio.02262-19.4 TABLE S4 Top differentially expressed pathways at 21 days postinfection. Shown are the most significantly represented pathways predicted at 21 dpi by Ingenuity Pathway Analysis (listed by lowest *P* value). Download Table S4, XLS file, 0.1 MB. Copyright © 2019 González et al. 2019 González et al. This content is distributed under the terms of the Creative Commons Attribution 4.0 International license.

IPA’s Upstream Regulator tool can predict the cascade of upstream factors that aid in understanding observed gene expression changes in a data set (i.e., FC differences) and help elucidate the biological activities occurring under the conditions being studied. At 7 dpi, the top predicted upstream elements include immune factors typical of bacterial infection such as LPS, IFN-γ, colony-stimulating factor 2 (CSF2), IL-6, STAT3, STAT1, TNF, and IL-1β (listed by *P* value in Table 2). These elements are similar at day 21, the exception being that the characteristic Th2 response markers IL-4, IL-10, and IL-13 are more significant (by *P* value in Table 2). This again can be interpreted as a shift from a Th1 response at 7 dpi to a Th2 response at 21 dpi.

**TABLE 2:** Top 25 IPA-predicted upstream regulators based on RNA-Seq data at 7 and 21 dpi^*a*^

Upstream regulator at 7 dpi	FC at 7 dpi	*P* value	Upstream regulator at 21 dpi	FC at 21 dpi	*P* value
LPS		8.07E−147	LPS		8.52E−58
IFN-γ	7.684	4.84E−119	IL-1β	3.163	3.70E−30
CSF2		1.23E−84	IL-4		3.71E−30
IL-6	4.096	2.07E−79	TNF		1.46E−29
STAT3	2.032	1.81E−74	Phorbol myristate acetate		2.58E−29
STAT1	11.417	1.48E−68	IFN-γ		3.20E−28
Poly(rI:rC) RNA		2.98E−68	IL-2		3.08E−26
TNF		2.44E−66	PPARA	−2.541	2.43E−24
E. coli B4 LPS		1.72E−65	IL-10		9.74E−24
IL-1β	7.953	8.01E−64	E. coli B5 LPS		9.94E−24
IL-10Rα	6.271	2.29E−62	TGF-β1	2.280	6.50E−23
IFN-α		3.30E−62	IL-10Rα	2.328	7.62E−22
IL-4		1.76E−61	IL-6		8.90E−21
TLR4		1.10E−57	IL-12 (complex)		3.59E−19
IRF7	9.472	3.78E−57	CSF2		5.27E−19
TLR3	2.260	4.59E−57	STAT3		1.85E−17
Dextran sulfate		1.52E−55	GATA2		8.65E−17
IFN-β1		3.18E−53	IL-13		1.07E−16
IFNAR1		5.96E−53	IL-21		3.40E−16
IL-10		1.33E−52	CEBPA		5.23E−16
IFNAR		4.87E−51	LEP		7.62E−16
TGF-β1	2.457	1.69E−50	E. coli B4 LPS		9.55E−16
IL-21		4.72E−50	STAT6		1.36E−15
PTGER4		1.12E−48	GATA3	5.807	3.52E−09

a Upstream regulators are ranked by *P* value (IPA). Fold change (FC) values for upstream regulators are from RNA-Seq data.

### Validation of RNA-Seq results.

NanoString technology was used to validate the transcriptome sequencing (RNA-Seq) results. NanoString measures RNA molecules directly in a reliable and sensitive manner without amplification or cloning, so no gene-specific biases are introduced (20). We used the nCounter mouse immunology panel, which targets 561 immunology-related mouse genes in order to analyze the gallbladder immune response. In general, fold changes from the top upregulated genes at both 7 and 21 dpi follow the same trends (see Table S5 in the supplemental material) as in the RNA-Seq experiment. For example, compare the changes at day 7 for *Tbx21* (NanoString FC = 15.86, RNA-Seq FC = 6.92), *Sh2d1a* (NanoString FC = 14.77, RNA-Seq FC = 3.97), and *Fcer1g* (NanoString FC = 12.73, RNA-Seq FC = 9.30) to those at day 21 for *Tbx21* (NanoString FC = 54.02, RNA-Seq FC = 3.60), *Sh2d1a* (NanoString FC = 27.12, RNA-Seq FC = 2.95), and *Batf* (NanoString FC = 11.3, RNA-Seq FC = 2.61).

10.1128/mBio.02262-19.5 TABLE S5 Comparison between differentially expressed genes at 7 and 21 dpi found using NanoString versus RNA-Seq. Download Table S5, DOCX file, 0.1 MB. Copyright © 2019 González et al. 2019 González et al. This content is distributed under the terms of the Creative Commons Attribution 4.0 International license.

### Immunohistochemistry shows an increase in overall B and T cells in lithogenic diet mice at 21 dpi.

**FIG 2:**
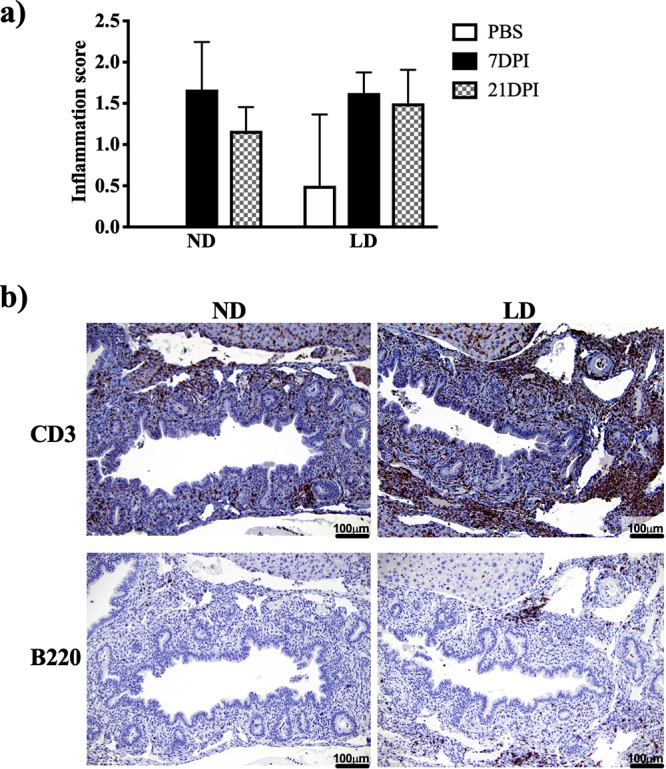
Despite similar levels of inflammation, *Salmonella*-infected lithogenic diet mice show an increased number of T and B cells in their gallbladders at 21 dpi with respect to normal diet mice. (a) Inflammation scores for gallbladders from mock-infected (PBS) and infected normal diet (ND) and lithogenic diet (LD) mice at 7 and 21 dpi. (b) Representative IHC images of mouse gallbladder tissue at 21 dpi for *Salmonella*-infected normal diet and lithogenic diet mice stained with anti-CD3 antibody (T cells) or anti-B220 (B cells). Magnification, 40×.

We have previously established that a lithogenic diet causes inflammation in the biliary tract of mice, even without *Salmonella* infection. This inflammation is characterized by a moderate influx of lymphocytes, macrophages, plasma cells, and neutrophils (13). Hematoxylin and eosin (H&E) staining was performed demonstrating no obvious differences in inflammation between normal diet (no gallstones) and lithogenic diet (with gallstones) mice at 7 or 21 dpi with *S.* Typhimurium (Fig. 2a). We then used immunohistochemistry to investigate whether we could observe a visible increase in T and B cells in the gallbladder of normal diet or lithogenic diet mice after *Salmonella* infection. Staining for CD3 revealed an enhanced recruitment of T cells in infected lithogenic diet versus normal diet (Fig. 2b). B220 staining showed a similar trend for B cells, although not as profuse as that observed with T-cells (Fig. 2b).

### *Salmonella*-infected lithogenic diet mice have increased numbers of lymphocytes and *Salmonella*-specific T cells in the gallbladder at 21 dpi.

**FIG 3:**
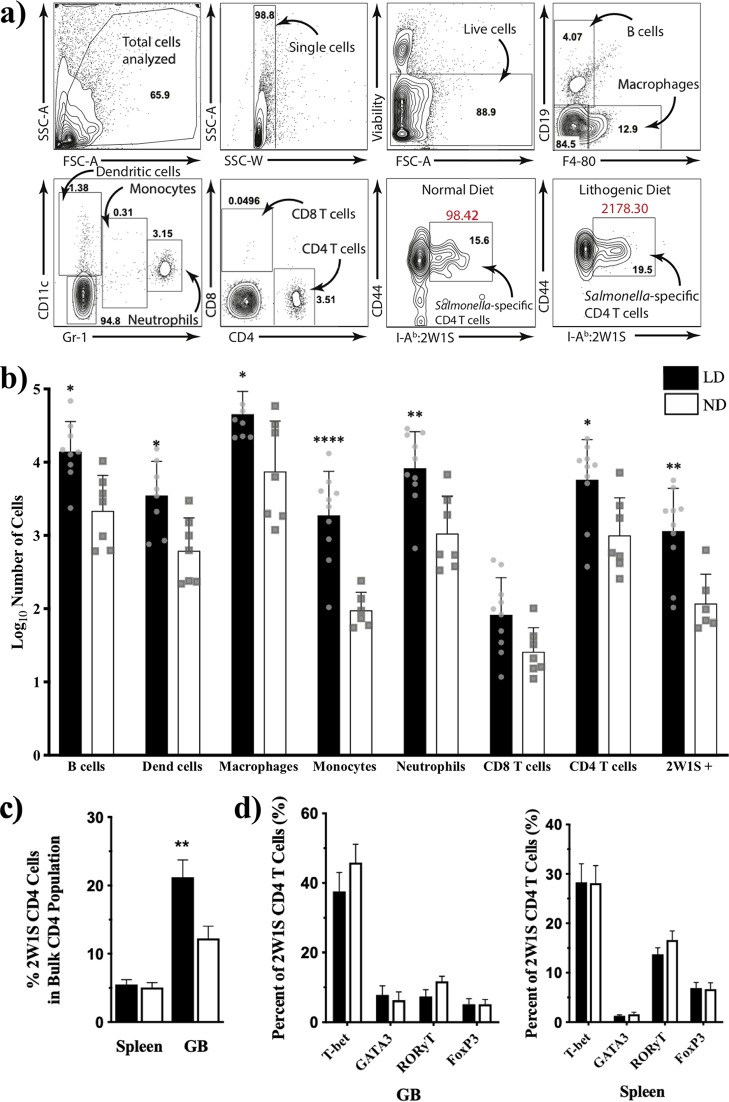
Lithogenic diet alters the number of lymphocytes in the gallbladder, but not the transcriptional profile of antigen-specific CD4^+^ T cells during *Salmonella* infection. Gallbladders (GB) and spleens were isolated from 129 × 1/SvJ mice (21 dpi) fed either a normal diet (ND) or lithogenic diet (LD). (a) Representative gates are shown for the gallbladder staining with each cell population labeled for clarity. Gallbladder cells were stained for viability, B cells (CD19), macrophages (F4/80), dendritic cells (CD11c), monocytes (CD11c-lo and Gr-1-lo), neutrophils (Gr-1-hi), and T cells (CD4 and CD8), as well as *Salmonella*-specific CD4^+^ T cells (CD4, CD44, and I-Ab-2W1S) from both groups of mice fed the normal and lithogenic diets. (b) Absolute cell numbers from gates in panel a were determined using cell counting beads, and the quantified total cell numbers are shown. (c and d) *Salmonella*-specific CD4^+^ T cells from spleen and gallbladder were fixed, permeabilized, and stained for intracellular transcription factors (T-bet, GATA-3, RORγt, and Foxp3). Analyses in panels a to d were completed on samples that were ≥50% viable by flow staining. In panel a, data from normal diet (*n* = 7) and lithogenic diet (*n* = 10) mice were log transformed and compared using 2-way ANOVA with Sidak *post hoc* testing. Data in panels b and c summarize two independent experiments (*n* = 18/group). In panel b, data were compared using an unpaired, two-tailed *t* test. In panel c, data were log transformed and compared using 2-way ANOVA with Sidak *post hoc* testing.

To gain a better understanding of the different immune cell populations in the gallbladder and to confirm our results that T and B cell populations increased at 21 dpi, we performed flow cytometry analysis of the spleens and gallbladders from mice infected with *S. Typhimurium* modified to express a well-characterized CD4 T cell epitope called 2W1S (21) (Fig. 3a). There was a significant difference in the number of immune cells between the two diets (*P* < 0.0001, 2-way analysis of variance [ANOVA]) with a significant increase in all cells analyzed (except CD8 T cells) in *Salmonella*-infected lithogenic diet mice (Fig. 3b). Consistent with RNA-Seq and immunohistochemistry, there was an increase in gallbladder B cells (*P* = 0.01). There was also a significant increase in the total numbers of CD4 T cells (*P* = 0.02). Importantly, this was reflected in a significant increase in the *Salmonella*-specific CD4 T cell population in the mice fed a lithogenic diet compared to normal diet when both groups were infected with 2W1S-tagged *S.* Typhimurium (*P* = 0.001) (Fig. 3b and c).

It was not clear why this increased population of *Salmonella*-specific T cells was unable to control infection in the lithogenic diet mice, so we sought to determine how these *Salmonella*-specific T cells might be phenotypically different between the two diets. We assessed the canonical Th transcription factors (T-BET for Th1, GATA3 for Th2, RORγT for Th17, and FOXP3 for regulatory T cells [Tregs]) in *Salmonella*-specific T cells in the spleen and gallbladder from infected mice fed with each diet (Fig. 3d). There was no significant difference in transcription factor expression between diets; however, due to the significant increase in total *Salmonella*-specific T cells in the lithogenic diet mice, as well as most other immune cells, it is likely that the inflammatory environment in the gallbladder of lithogenic diet mice is greater than that of normal diet mice.

## DISCUSSION

As expected, RNA-Seq data show a very strong immune response at 7 dpi, with an abundance of cytokines, chemokines, and other immune response-related genes (Table 1; Table S1). The most highly upregulated gene in *Salmonella*-infected lithogenic diet mice was *Chil1*, which codes for an inactive chitinase. Interestingly, CHI3L1 (an ortholog of CHIL1) has been shown to enhance Serratia marcescens adhesion to epithelial cells (16), and because we have previously shown that *Salmonella* can adhere to and aggregate on mouse gallbladder epithelial cells (14), this suggests that CHI3L may play a role during chronic *Salmonella* gallbladder infection. Remarkably, at 21 dpi most of the upregulated genes coded for immunoglobulins. This phenomenon is observed in other chronic biofilm infections, such as cystic fibrosis, where the antibody response is unable to clear the infection, which results in the accumulation of immune complexes that can damage tissues. (22). Previous studies using a *Salmonella*-resistant mouse strain have shown that a strong antibody response is important for protective immunity against *Salmonella* (23). Additionally, studies in African children have demonstrated the essential role of antibodies in killing blood-borne extracellular nontyphoidal strains of *Salmonella* (NTS), where cell-mediated immunity does not provide protection (24, 25).

Many of the most highly downregulated genes at 7 and 21 dpi corresponded to hormone metabolism (Table S1 and Table S3). Interestingly, this contradicts a previous metabolomics study that found that steroid metabolism was highly upregulated in the liver and in fecal samples following *Salmonella* infection (26). This could again highlight the unique gallbladder conditions during cholelithiasis that allow *Salmonella* to persist, and future studies should focus on this important aspect of gallbladder colonization. Furthermore, it is well documented that women are more prone to be chronic carriers: this is mostly believed to be a result of women also having a higher incidence of cholelithiasis (27, 28). However, it has been suggested that female susceptibility to typhoid could be due to estrogens, as treatment with estradiol or progesterone either increases or decreases female mouse susceptibility after challenge with intraperitoneally (i.p.)-injected *S.* Typhimurium (29, 30). The fact that these genes consistently appeared among the downregulated loci in our RNA-Seq experiments allows us to hypothesize that these hormones could be playing a role during chronic carriage.

NanoString was used as a validation method for RNA-Seq. Although this technique does not give a global picture of gene expression like RNA-Seq, fold changes from the top upregulated genes at both 7 and 21 dpi follow the same trends (Table S5) as the RNA-Seq experiment. Dissimilarities in fold changes could be explained by the fact that RNA was isolated from the gallbladder of different animals than those used for RNA-Seq, as the immune response are likely sensitive to bacterial burden and the kinetics of biofilm formation in the gallbladder, which can be variable between animals.

The main objective of this study was to shed light on the conditions that allow *Salmonella* to persist in the gallbladder. *Salmonella* has been shown to persist within macrophages in mesenteric lymph nodes for as long as 1 year in *Slc11a1*^+/+^ (*Nramp*^+/+^) mice (31), preferentially associating with anti-inflammatory/M2 macrophages (32). *Salmonella* has also been shown to persist inside hemophagocytic macrophages (33) and to induce macrophages to phagocytose erythrocytes (34). Additionally, dormant *Salmonella* cells have also been found to persist within macrophages of susceptible mice (35). Unlike carriage within macrophages, which follows classic intracellular *Salmonella* infection, the chronic carrier state associated with colonization of the biliary tract involves the formation of a biofilm. Because of this, we hypothesized that chronic *Salmonella* carriage will display many of the characteristics of other biofilm-associated chronic diseases. One of these signature features displayed by various chronic biofilm infections is the skewing of the immune response toward either a proinflammatory or anti-inflammatory pathway depending on the pathogen (12, 22, 36). Our RNA-Seq data display a switch from a Th1 to a Th2 immune response given the abundance of immunoglobulins and the presence of the transcriptional regulator gene *Gata3* at 21 dpi versus the marked proinflammatory response observed at 7 dpi (Table S1 and Table S2). IPA also indicates an increased importance of Th2 regulators like IL-4 (Table 2). Although not in the context of a biofilm, this phenomenon has been previously observed in chronic S. enterica serovar Enteritidis carriage in chickens where enterocytes isolated from susceptible chickens showed a Th2 bias (37) and in S. enterica serovar Pullorum persistent infection, where the pathogen was recently shown to drive host immunity from a Th17 toward a Th2-like response (38). Interestingly, IPA also identified several T-cell-related pathways at 21 dpi (Table S4). This piece of data led us to investigate whether there was a difference in the endogenous, *Salmonella*-specific T cell responses in our chronic carriage mice fed a lithogenic diet with gallstones versus normal diet mice with no gallstones. Immunohistochemistry qualitatively showed an increased abundance of T and B cells in *Salmonella*-infected lithogenic diet mice versus normal diet mice. Flow cytometry analysis also showed a significant increase in the number of lymphocytes (except CD8 T cells) in the infected lithogenic diet mice (Fig. 3b). However, we did not find a significant difference in the profile of transcriptional regulatory factors between normal diet and lithogenic diet mice (Fig. 3d). The RNA-Seq experiments compared infected lithogenic diet mice to mock-infected lithogenic diet mice (as opposed to infected normal diet versus infected lithogenic diet mice), which might explain why we saw an upregulation of *Gata3* at 21 dpi. Intracellular transcription factor staining did show a higher percentage of GATA3-expressing CD4 T cells in the gallbladder than in spleen, which suggests that the gallbladder microenvironment is more anti-inflammatory.

It is notable that all mice on the lithogenic diet demonstrated higher levels of inflammation at 21 dpi in the gallbladder compared to mice on a normal diet (Fig. 2a), yet upon infection were incapable of bacterial clearance. While this inflammation included cells such as macrophages and dendritic cells (Fig. 3b), more intriguing was the observation that the numbers of *Salmonella*-specific CD4 T cells were significantly higher in the gallbladders of infected mice on the lithogenic diet (Fig. 3c). While it is known that CD4 T cells are essential mediators of anti-*Salmonella* immunity in lymphoid tissues such as the spleen and mesenteric lymph nodes, less is known about the role of these cells in the hepatobiliary system, specifically the gallbladder. Here represents the first instance where endogenous, *Salmonella*-specific CD4 T cells have been observed to migrate into the gallbladder in response to increased local infection. While most of these cells expressed T-bet, the canonical Th1 transcription factor, Th17 and Treg cells were also represented in these *Salmonella*-specific T cells (Fig. 3d). It is known that Th1 cells, and particularly the Th1 cytokine IFN-γ, are important for control or elimination of persistent *Salmonella* infection. Our data imply that these T cells should potentiate eradication of the bacteria; however, this was not the case. One possible explanation for this is that the Treg cells in the gallbladder are controlling Th1 T cell inflammation, thus preventing bacterial clearance. It has been shown that ablation of Tregs early after infection increased the effectiveness of Th1 responses and controlled the promptness of persistent *S.* Typhimurium infection (39). The increase in IL-10 mRNA transcripts in the gallbladder from 7 to 21 dpi supports this possibility. While we have previously demonstrated that the the gallbladder—particularly the gallbladder-housed cholesterol gallstones—provides a protective niche for bacterial persistence, it was not previously clear whether it was immune privileged. Here we show that the gallbladder is, in fact, readily infiltrated by anti-*Salmonella* immune cells that are incapable of eliminating this niche. Perhaps this cholesterol-rich environment facilitates bacterial survival and allows for the continuous reseeding of the gut lumen, where bacteria can then be shed in the feces despite the presence of antibacterial T cells. Future studies will more carefully assess the role of each of these immune cell types in the gallbladders of these mice.

## MATERIALS AND METHODS

### Ethics statement.

Mouse care and housing was carried out in accordance with guidelines established by the Ohio State University (OSU) Institutional Animal Care and Use Committee (IACUC). Animal work was previously approved by OSU IACUC. The Ohio State University Animal Care and Use Program is accredited by the Association for the Assessment and Accreditation of Laboratory Animal Care International (AAALAC). Research activities conformed to the statutes of the Animal Welfare Act and guidelines of the Public Health Service as required in the *Guide for the Care and Use of Laboratory Animals* (40).

### Bacterial strains and growth conditions.

*S.* Typhimurium strain 14028 OmpC was genomically tagged with the T cell epitope 2W1S (EAWGALANWAVDSA), as previously described using primers designed with extension arms homologous to the portion of the OmpC gene, deleting the stop codon, and extending downstream from it and then utilizing the lambda red system for recombination (21). *S.* Typhimurium strains 14028 (JSG210) and JSG4027 (14028 OmpC-2W1S) were streaked on Luria-Bertani (LB) agar plates and incubated at 37°C overnight. Single colonies were used to start overnight liquid cultures. Planktonic cells were grown at 37°C on a rotating drum in LB. The growth medium for JSG4027 was supplemented with kanamycin (40 μg ml^−1^).

### Murine model of typhoid carriage.

Thirty-six male 129 × 1/SvJ mice were fed a lithogenic diet (1% cholesterol and 0.5% cholic acid (Envigo/Harlan Laboratory, IN, USA) for 8 weeks (4): half were injected intraperitoneally (i.p.) with PBS (mock infection control), and half were inoculated i.p. with a dose of 2 × 10^4^ CFU of *S.* Typhimurium (Fig. 1a). Half the mice from each group were sacrificed at 7 dpi and the remaining half at 21 dpi. At both time points, gallbladders were removed and flash frozen in dry ice for RNA extraction. For immunohistochemistry and flow cytometry experiments, age- and sex-matched normal diet mice were used as controls.

### RNA extraction.

For each of the four biological replicates, we pooled three gallbladders, suspended in 1 ml TRIzol and homogenized in a TissueLyser LT (Qiagen, Valencia, CA, USA) for 10 min at 50 Hz. RNA was isolated using the RNeasy minicolumn kit (Qiagen, Valencia, CA, USA) following the manufacturer’s instructions. RNA quality was assessed using a BioAnalyzer 2100 with a Eukaryote Total RNA Nano Chip (Agilent, Palo Alto, CA, USA).

### RNA-Seq analysis.

For each sample, read pairs were trimmed to remove sequencing adapters and aligned to the GRCm38.p4 assembly of the mouse reference from NCBI using version 2.5.1b of the RNA-Seq aligner STAR (41). Features were identified from the GFF file that came with the assembly from NCBI. Feature coverage counts were calculated using HTSeq (42). Differentially expressed features were calculated using DESeq2 (43) and custom scripts developed in-house to perform RNA-Seq analysis. Functional analysis was performed on differentially expressed genes in each time point and were uploaded and analyzed separately using the software package IPA (Ingenuity Systems, Redwood City, CA, USA). The resulting biological functions, canonical pathways, and upstream regulators were filtered by setting a threshold of *P* < 0.05 using Fisher’s exact test.

### NanoString.

We used the nCounter mouse immunology panel (NanoString Technologies, Seattle, WA, USA), which targets 561 immunology-related mouse genes. A total of 200 ng of RNA was hybridized overnight with nCounter reporter probes in hybridization buffer and in excess of nCounter capture probes at 65°C for 16 to 20 h. After overnight hybridization, excess probes were removed using two-step magnetic bead-based purification on an automated fluidic handling system (nCounter Prep Station). Biotinylated capture probe-bound samples were immobilized and recovered on a streptavidin-coated cartridge. The abundance of specific target molecules was then quantified using the nCounter digital analyzer. Individual fluorescent barcodes and target molecules present in each sample were recorded with a charge-coupled device (CCD) camera by performing a high-density scan (600 fields of view). Images were processed internally into a digital format and exported as a reporter code count (RCC). Data were analyzed using nSolver software provided by NanoString Technologies.

### IHC.

Mice that had been fed either normal diet or lithogenic diet were inoculated with either PBS or *S.* Typhimurium. After 7 or 21 dpi, gallbladders were removed and fixed in 10% neutral buffered formalin phosphate (Fisher Scientific, MA) for 72 h. Fixed tissues were then embedded in paraffin wax. Samples were treated and stained as previously described for hematoxylin and eosin (H&E) (13). For immunohistochemistry (IHC), paraffin sections were deparaffinized with xylene and graded ethanols, hydrated in distilled water, and stained using T-cell-specific (anti-CD3) or B-cell-specific (anti-B220) antibodies by the OSU Comparative Pathology and Mouse Phenotyping Shared Resource.

### Tissue treatment.

Mouse gallbladder and spleen cells were isolated by mechanical dissociation, followed by a 1-h enzymatic digestion at 37°C with 1 mg/ml collagenase XI (Sigma) in Dulbecco’s modified Eagle’s medium (DMEM) containing 10% fetal bovine serum (FBS) and 4 mM l-glutamine. Following digestion, cells were filtered through a 100-μm mesh screen, centrifuged, then washed and resuspended in sorter buffer (2% newborn calf serum [NCS], 0.1%NaN_3_, and 2 mM EDTA in PBS) for subsequent flow staining.

### Flow cytometry.

Cells were stained with commercially available antibodies for assessment by flow cytometry. The following antibodies were used. Anti-CD11c (clone N418), anti-CD11b (clone M1/70), anti-F4/80 (clone BM8.1), anti-CD19 (clone 1D3) in vFluor450, anti-CD4 labeled with fluorescein isothiocyanate (FITC [clone RM4-5]), anti-CD3ε labeled with peridinin chlorophyll protein (PerCP)-Cy5.5 (clone 145-2C11), Ghost Dye 780 (viability), and purified anti-mouse CD16/CD32 (Fc Block [clone 2.4G2]) were purchased from Tonbo Biosciences (San Diego, CA). Anti-CD4 (clone RM4-5) and anti-F4/80 (clone BM8) in BV510, anti-CD44 labeled with phycoerythrin (PE [clone IM7]), anti-CD8a AF700 (clone 53-6.7), anti-CD44 PerCP-Cy5.5 (clone IM7), anti-CD19 (clone 6D5), anti-T-bet (clone 4B10), and anti-mouse IgG1κ (clone MOPC-21) in BV605 and anti-Foxp3 AF488 (clone MF-14) were purchased from BioLegend (San Diego, CA). Anti-GATA3 PE-Cy7 (clone TWAJ) and anti-mouse IgG1κ AF488 (clone P3.6.2.8.1) were purchased from Invitrogen. Anti-RoRγt (clone AFKJS-9) and anti-rat IgG1 (clone eBRG1) in PE and anti-rat IgG1 PE-Cy7 (clone eBRG1) were purchased from eBioscience. Anti-GR-1 PE-Cy7 (clone RB6-8C5) was purchased from BD Pharmingen (Pont de Claix, France). For all surface and intracellular staining protocols, cells were blocked with Fc Block mixed with 2% mouse and rat serum for 10 min on ice and then surface stained at 1:100 dilutions unless otherwise indicated. Samples were collected using a BD Biosciences LSRII Fortessa cytometer. All fluorescence-activated cell sorting data were analyzed with FlowJo software v9.9.3 (FlowJo, LLC, Ashland, OR).

### Intracellular staining.

Cells were blocked with Fc Block and stained with 10 nM MHC class II tetramer (I-A^b^ 2W1S-allophycocyanin [APC]) in sorter buffer for 1 h at room temperature. Cells were next stained for viability in azide/serum-free PBS for 30 min at 4°C, followed by surface staining in sorter buffer at 1:100 dilutions for 30 min at 4°C with anti-CD11b, CD11c, CD19, F4/80, GR-1, CD3ε, CD4, CD8a, and CD44. For intracellular transcription factor staining, cells were fixed, permeabilized, and subsequently stained for the transcriptional factors or the appropriate isotype control using the Foxp3 staining buffer kit (eBioscience).

### Statistical analysis.

For flow cytometry experiments, total cell counts were log transformed and compared using a 2-way ANOVA with Sidak *post hoc* testing or a two-tailed *t* test with the statistical software GraphPad Prism version 7.0e. Statistical significance was represented as follows: *, *P* ≤ 0.05; **, *P* ≤ 0.01; ***, *P* ≤ 0.001; and ****, *P* ≤ 0.0001.

### Accession number(s).

The RNA sequencing information has been deposited in the Gene Expression Omnibus (GEO) Sequence Read Archive of the National Center for Biotechnology Information (https://www.ncbi.nlm.nih.gov/geo/) under accession no. GSE136893 (https://www.ncbi.nlm.nih.gov/geo/query/acc.cgi?acc=GSE136893).
