# Supplementary material for: Establishment of Chronic Typhoid Infection in a Mouse Carriage Model Involves a Type 2 Immune Shift and T and B Cell Recruitment to the Gallbladder
Source: mBio. 2019 Oct 1;10(5):e02262-19. doi: 10.1128/mBio.02262-19 (PMC6775463; doi:10.1128/mBio.02262-19)
Supplement: TABLE S5 [file mBio.02262-19-st005.docx]

**Table S5.** Comparison between differentially expressed genes found using nanostring vs. RNA-Seq

| **7DPI** | **Nanostring** | | **RNA-Seq** | |
| --- | --- | --- | --- | --- |
| **Gene** | **FC** | **P value** | **FC** | **P value** |
| TBX21 | 15.86 | 5.70E-03 | 6.92 | 1.44E-08 |
| PSMB8 | 14.77 | 2.00E-04 |  |  |
| SH2D1A | 12.73 | 1.74E-02 | 3.97 | 2.57E-04 |
| FCER1G | 11.57 | <000.1 | 9.30 | 5.11E-15 |
| TAP1 | 11.46 | 1.20E-03 |  |  |
| IRF1 | 9.86 | 3.50E-03 | 6.95 | 6.26E-12 |
| SLC2A1 | 9.53 | 9.20E-03 |  |  |
| BATF | 9.02 | 1.58E-01 | 15.48 | 3.55E-16 |
| SOCS1 | 8.94 | 8.22E-02 | 7.95 | 3.12E-09 |
| IRF8 | 7.49 | 5.40E-03 | 6.84 | 3.26E-15 |
| NCF4 | 7.29 | 1.68E-02 | 6.88 | 4.58E-09 |
| CTLA4-TM | 6.8 | 2.53E-02 |  |  |
| POU2F2 | 4.16 | 1.42E-02 | 3.55 | 6.83E-04 |
|  |  |  |  |  |
| **21DPI** | **Nanostring** | | **RNA-Seq** | |
| **Gene** | **FC** | **P value** | **FC** | **P value** |
| TBX21 | 54.02 | 5.00E-04 | 3.60 | 3.31E-03 |
| SH2D1A | 27.12 | 9.00E-04 | 2.95 | 5.99E-03 |
| BATF | 11.3 | 7.96E-02 | 2.61 | 6.75E-02 |
| PSMB8 | 10.51 | 1.00E-04 |  |  |
| SOCS1 | 9.62 | 6.40E-03 |  |  |
| TAP1 | 9.45 | 5.50E-03 |  |  |
| BTK | 8.96 | 3.21E-02 | 2.10 | 9.14E-02 |
| FCER1G | 6.53 | 3.00E-03 |  |  |
| HLA-DRB3 | 6.31 | 1.25E-02 |  |  |
| IRF1 | 5.75 | 1.90E-03 |  |  |
| IRF8 | 5.24 | 4.00E-04 |  |  |
| ZAP70 | 4.27 | 3.83E-02 | 3.37 | 2.17E-03 |
| POU2F2 | 4.24 | 6.30E-03 | 2.87 | 2.46E-03 |
